# A well-preserved ‘placoderm’ (stem-group Gnathostomata) upper jaw from the Early Devonian of Mongolia clarifies jaw evolution

**DOI:** 10.1098/rsos.221452

**Published:** 2023-02-22

**Authors:** Martin D. Brazeau, Haobo Yuan, Sam Giles, Anna L. Jerve, E. Zorig, Ya. Ariunchimeg, Robert S. Sansom, Robert C. Atwood

**Affiliations:** ^1^ Department of Life Sciences, Imperial College London, Silwood Park Campus, Ascot SL5 7PY, UK; ^2^ The Natural History Museum, London SW7 5BD, UK; ^3^ School of Geography, Earth and Environmental Sciences, University of Birmingham, Birmingham B15 2TT, UK; ^4^ Institute of Paleontology, Mongolian Academy of Sciences, Ulaanbaatar 15160, Mongolia; ^5^ The Natural History Museum, Ulaanbaatar 1420, Mongolia; ^6^ Department of Earth and Environmental Sciences, University of Manchester, Manchester M13 9PT, UK; ^7^ Diamond Light Source, Didcot OX11 0DE, UK

**Keywords:** acanthothoracid, placoderm, Devonian, jaws, Mongolia, evolution

## Abstract

The origin of jaws and teeth remains contentious in vertebrate evolution. ‘Placoderms’ (Silurian-Devonian armoured jawed fishes) are central to debates on the origins of these anatomical structures. ‘Acanthothoracids’ are generally considered the most primitive ‘placoderms’. However, they are so far known mainly from disarticulated skeletal elements that are typically incomplete. The structure of the jaws—particularly the jaw hinge—is poorly known, leaving open questions about their jaw function and comparison with other placoderms and modern gnathostomes. Here we describe a near-complete ‘acanthothoracid’ upper jaw, allowing us to reconstruct the likely orientation and angle of the bite and compare its morphology with that of other known ‘placoderm’ groups. We clarify that the bite position is located on the upper jaw cartilage rather than on the dermal cheek and thus show that there is a highly conserved bite morphology among most groups of ‘placoderms’, regardless of their overall cranial geometry. Incorporation of the dermal skeleton appears to provide a sound biomechanical basis for jaw origins. It appears that ‘acanthothoracid’ dentitions were fundamentally similar in location to that of arthrodire ‘placoderms’, rather than resembling bony fishes. Irrespective of current phylogenetic uncertainty, the new data here resolve the likely general condition for ‘placoderms’ as a whole, and as such, ancestral morphology of known jawed vertebrates.

## 1. Introduction

‘Placoderms’ are extinct fishes that ranged from the Silurian to the end of the Devonian period (444 to 359 Ma). Although their monophyly is highly debated [1–5], it is widely agreed that they are the only known jaw-bearing stem-group gnathostomes. They are thus key to reconstructing character transitions leading to modern (crown-group) gnathostomes. ‘Placoderms’ occupy a central role in debates on the origin of both jaws and teeth—major gnathostome innovations that led to diverse feeding ecologies. Recent discoveries from the Silurian and Early Devonian of south China [4,6,7] and Czechia [8] have delivered significant advances that diminish the morphological gap between ‘placoderm’ and crown-group vertebrate jaw morphology. There is now a growing consensus that the last common ancestor of crown gnathostomes possessed dermal jaw bones, a trait potentially extending to the earliest appearance of jaws [9]. This has transformed perspectives of both jaw and tooth evolution, which have long been modelled on shark-like conditions [10].

Owing to a lack of fossilized mandibular arch material in jawless fishes, the primitive structure of the gnathostome jaw has been difficult to reconstruct using palaeontological evidence. ‘Placoderms’ exhibit diverse jaw morphologies and presumed feeding ecologies [11–13]. These range from diverse biting modes, suspension feeding and possible grazing in arthrodires; durophagy in ptyctodonts; ambush predation in rhenanids; and benthic (detritus?) feeding in antiarchs. Most ‘placoderm’ dentitions were relatively simple: tubercles added in rows (or files) or centripetally to growing jaw plates [14,15]. Despite this simplicity, ‘placoderm’ dentitions are diverse, and detailed anatomical investigations in recent decades have highlighted similarities between some ‘placoderm’ groups (such as arthrodires) and crown-group gnathostomes [14,16]. More recently, it has been proposed [8] that some ‘acanthothoracids’ bore marginal teeth aligned in rows along the jaw edge, as in most crown-group gnathostomes. Understanding the relationship of ‘placoderm’ dentitions as well as overall jaw morphology and functional diversity are thus key to understanding the origin of modern jaws and teeth.

Here we describe a nearly complete, fully three-dimensional upper jaw (palatoquadrate and suborbital plate) of an ‘acanthothoracid’ from the Early Devonian (Pragian) of Mongolia. The specimen reveals the most complete quadrate (articular condyle connecting the lower jaw) of any ‘acanthothoracid’ known to date. This allows us to reconstruct and compare the orientation and angle of the bite in these ‘placoderms’, and we show that a highly similar jaw articulation and bite occurs between three groups of ‘placoderms’ with disparate presumed ecomorphologies: ‘acanthothoracids’, rhenanids and arthrodires, suggesting conservation of a shared ancestral morphology among these groups. Furthermore, we clarify the evolution of gnathostome dentitions by revising the interpretation of unusual dental morphology in a previously described ‘acanthothoracid’.

## 2. Material and methods

### 2.1. Specimens

The upper jaw described here occurs within a bedrock sample MPC-Fh200/10.4 from the Yamaat Gol locality in western Mongolia [17–19]. The absence of endochondral bone and the tight spacing of the tubercles indicate that this palatoquadrate does not belong to *Minjinia*, the only ‘placoderm’ taxon so far named from that locality. The Yamaat Gol fauna contains at least three distinct ‘acanthothoracid’ taxa, but all are based on isolated fossils, making taxonomic assignments fraught. This paper makes no further taxonomic attribution for the specimen as it lacks other diagnostic traits.

### 2.2. Synchrotron tomography

We performed synchrotron X-ray micro-computed tomography at the I12 beamline of the Diamond Light Source, United Kingdom [20]. The X-ray beam was set to a monochromatic energy of 90 keV (double bent Laue Si 111 monochromator). The regions of interest were scanned in I12 Experimental Hutch One using the beamline's modular imaging system. This detector consists of a PCO.edge 5.5 sCMOS camera and four user-selectable optical modules, each comprising a scintillator, 90-degree turning mirrors and a visible light lens. We used module 2 with a magnification of 0.820, corresponding to a recorded pixel size of 7.91 µm. Each acquisition consisted of 1800 projections, of 15 ms exposure time each, over a 180° rotation of the sample. Additionally, 50 flatfield images (sample out of the beam) were recorded before and after the series of acquisition as well as 10 dark images (X-ray beam off to record the noise of the camera).

The tomographs were reconstructed using the SAVU tomographic processing software [21,22] developed at Diamond Light Source. In the reconstruction process, ring artefact removal [23] and auto-centring [24] were applied, as well as distortion correction [25]. The low-pass filter approach [26] was applied. Filtered back-projection reconstructions were performed using the Astra library [27,28], and the whole process was applied on an HPC cluster system using the SAVU tomography pipeline.

### 2.3. Data segmentation and visualization

We segmented the data using Materialise Mimics version 23.0 (https://www.materialise.com/en/healthcare/mimics-innovation-suite/mimics; Materialise, Leuven, Belgium). Three-dimensional models were exported and rendered in Blender v.3.2.2 (Blender Foundation; https://www.blender.org).

### 2.4. Phylogenetic analysis

We reproduced the analysis of Vaškaninová *et al*. [8] with modified scorings for *Radotina* to reflect uncertainties and interpretations presented here. A full list of score changes is provided in electronic supplementary material, table S1. We restricted our data re-codings to eight characters relating to the identification of teeth and sensory canals in *Radotina*. We analysed the matrix using TNT (v. 1.5) [29] using a ‘new technology search’ (xmult) set to level 10 including the parsimony ratchet with 50 replicates. We then applied branch-swapping (bbreak) to the trees in memory. To analyse the distribution of character states, we generated a fully resolved topology removing ‘wildcard’ taxa by generating an agreement subtree in PAUP* (v. 4.0 alpha test version build 168) [30]. We visually explored ancestral states conditions for jaw characters using parsimony with the ‘Trace Character History’ function in Mesquite (v. 3.70) [31].

## 3. Results

### 3.1. Description

**Figure 1.**
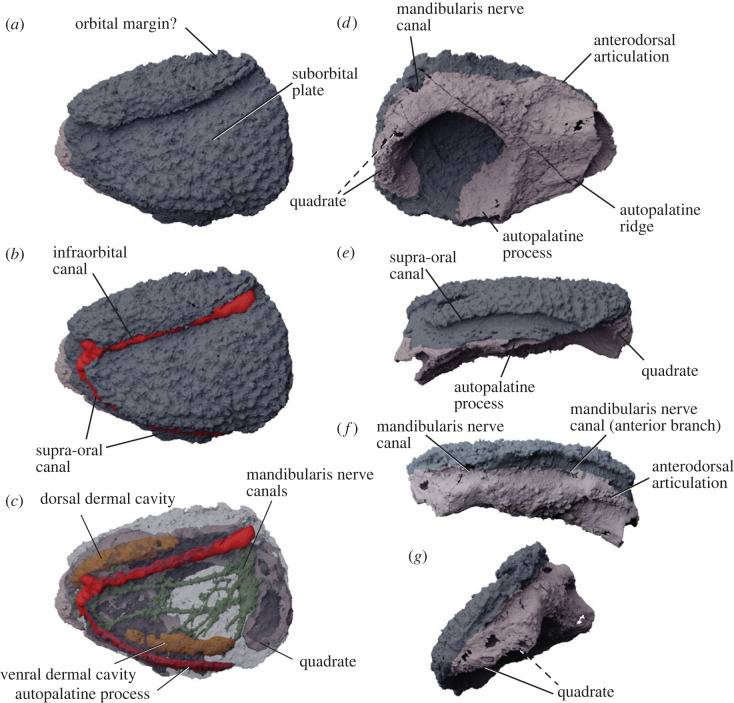
MPC-Fh200/10.4 upper jaw complex in virtual three-dimensional rendering from synchrotron tomography. (*a*) Lateral view; (*b*) lateral view with in-fill of lateral line canals (red); (*c*) lateral view with suborbital plate rendered semi-transparent to reveal internal cavities and canals; (*d*) mesial view; (*e*) ventral (labial) view; (*f*) dorsal view; (*g*) posterior view. Blue-grey indicates suborbital (dermal) plate; mauve indicates perichondral sheath of palatoquadrate. Exact boundaries between these units are approximate. Dashed leader line indicates uncertain attribution.

**Figure 2.**
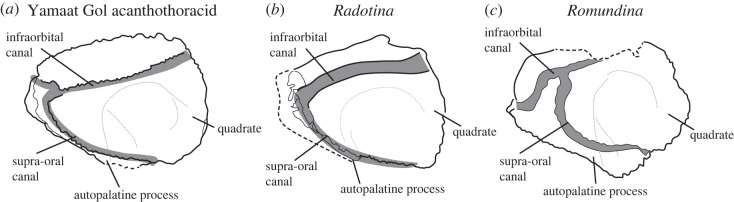
(*a*) Lateral line canal pattern on the cheek of ‘acanthothoracid’ placoderms showing the position of the canals relative to the palatoquadrate (dotted line). (*b*) Drawing based on data from [8]; (*c*) interpretive drawing of specimen from [32].

MPC-Fh200/10.4 is a nearly complete upper jaw unit consisting of the suborbital plate and palatoquadrate ossification (figure 1). The palatoquadrate is preserved as a thin shell of perichondral bone; endochondral bone is absent. The specimen is mostly undeformed, retaining its original three-dimensional shape. The suborbital plate is covered in densely placed stellate tubercles. In lateral view, the suborbital plate has a geometry similar to *Radotina* (figure 2*b*): the outline is roughly trapezoidal; the posterior margin is deep (most of the depth of the plate) and slightly convex. There is no evidence of an orbital process on the dorsal surface, but we cannot rule out the possibility that it was worn or broken off. As in *Radotina*, the ventral margin of the suborbital plate is gently convex. This differs from *Romundina*, where the ventral profile has a marked angle between the autopalatine (anterior) portion and the adductor fossa area, contributing to a short posterior margin [32,33]. The most prominent feature of the external face of the suborbital is the infraorbital canal, which follows a course from the posterodorsal corner to the anterior margin of the plate. The upper margin of the plate forms a pronounced overhang of the infraorbital sensory canal. As in *Radotina*, the dorsal tubercles curl over the dorsal margin of this canal. The anterior end of the infraorbital canal curves downward to meet the supra-oral sensory canal. The latter traces the preserved ventral margin of the suborbital. This canal terminates between the autopalatine process and the quadrate.

In medial view, the palatoquadrate fills the perimeter of the suborbital plate. The surface of the perichondral bone in the virtual models has a nodular texture. However, these appear to be ferrous crystals formed on the surface. The palatoquadrate has the classic ‘placoderm’ omega shape, forming a thick arch around an almost circular adductor muscle chamber. The geometry and proportions of the palatoquadrate and adductor fossa again closely resemble *Radotina* [8]. The adductor fossa occupies about half of the internal face. The autopalatine is commensurately short and turns medially at its anterior end. The ossification is well preserved, showing that it ended anteriorly with a single, open articulation facet as in rhenanid ‘placoderms’ [34]. On the dorsal side of the autopalatine is a low, blunt process, which we interpret as an anterodorsal articulation (figure 1*d*).

Exceptionally among ‘acanthothoracid’ fossils, the quadrate area is almost entirely preserved (figure 1*d–f*). For the first time, it allows a complete understanding of the jaw articulation in these ‘placoderms’. The quadrate is mediolaterally broad and possibly double-headed as in osteichthyans. Slightly dorsal and anterior to the quadrate, a large-diameter canal pierces the element between the dermal bone and the palatoquadrate. This canal corresponds to the mandibularis canal (nerve V) described in *Holonema* [35]; a similar canal is seen but undescribed in *Radotina* [8]. Further anterior along the dorsal side of the palatoquadrate, the top of the palatoquadrate is pierced by an oblique, anteriorly directed canal similar to one seen in *Radotina* (figure 1*f*, ‘mandibularis nerve canal (anterior branch)’). These canals branch into a multitude of ramules in the autopalatine cartilage (figure 1*c*).

The internal structure of the bone is relatively well preserved. Three rami of the mandibularis branch are preserved. These extend to the labial margin of the suborbital plate. Two extend in a nearly parallel course from the trunk canal in the posterior quarter of the plate all the way to the antero-labial corner, where fine ramules underly the junction of the supra-oral and infraorbital sensory canal. The suborbital plate of MPC-Fh200/10.4 carries short, buried dorsal and ventral cavities that parallel the sensory canals. The chambers do not have any apparent opening to the outside. We are not aware of any such structures in any other ‘placoderms’, but suggest they are homologues of the ‘cutaneous sensory openings' seen in various ‘placoderms’ [36]. Such canals have not been observed in the suborbital plates of any ‘acanthothoracids’ so far, but no similarly complete examples have been studied by computed tomography scanning. Nevertheless, a cutaneous sensory opening extends parallel to the infraorbital canal in *Romundina* immediately posterior to the orbital margin (see Ørvig [32]; Dupret *et al*., [37], termed ‘sensory pits’ by the latter). Examination of the tomograms by Dupret *et al*. confirms that the sensory pits are connected to a tubular cavity.

### 3.2. Phylogenetic analysis

The phylogenetic analysis resulted in 16 800 trees with a score of 947 steps (see electronic supplementary material, information). The strict consensus matches the topology recovered by Vaškaninová *et al*., placing the ‘acanthothoracids’ as an unresolved assemblage joining at the base of mandibulate stem-group gnathostomes alongside antiarchs. However, the agreement subtree shows that the lack of resolution in this part of the tree was attributable to the highly incomplete, unnamed acanthothoracid snout from Drake Bay, referred to only by its specimen number CPW.9, and *Brindabellaspis*. Neither of these taxa is represented by mandibular arch material. The removal of these two taxa reveals an acanthothoracid clade immediately crownward of antiarchs. To view synapomorphies, we used the describetrees function in PAUP*. This clade is supported by two unambiguous synapomorphies according to this dataset (character numbers derive from the dataset of Qiao *et al*. [38]): character 97: the presence of a ventral notch between the parachordals (consistency index: 0.125); and character 103: the absence of complete dermal encirclement of the pectoral fin base (consistency index: 0.167).

## 4. Discussion

### 4.1. Comparative anatomy and function of upper jaws in ‘placoderms’

**Figure 3.**
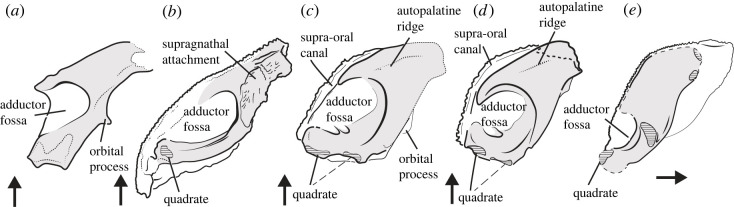
Upper jaws of placoderm fishes in internal view. (*a*) *Jagorina* (based on Museum für Naturkunde, Berlin, specimen MB.f.510.5); (*b*) Buchanosteid based on data published in [39]; (*c*) *Radotina* based on surface models from [8]; (*d*) MPC-Fh200/10.4 (original); (*e*) *Bothriolepis* (after [40]). Arrow indicates anterior. Horizontally hatched areas indicate articulation facet. Dashed label lines indicate uncertainty concerning facet as part of quadrate or possible hyoid attachment. Dashed leader line indicates uncertain attribution.

**Figure 4.**
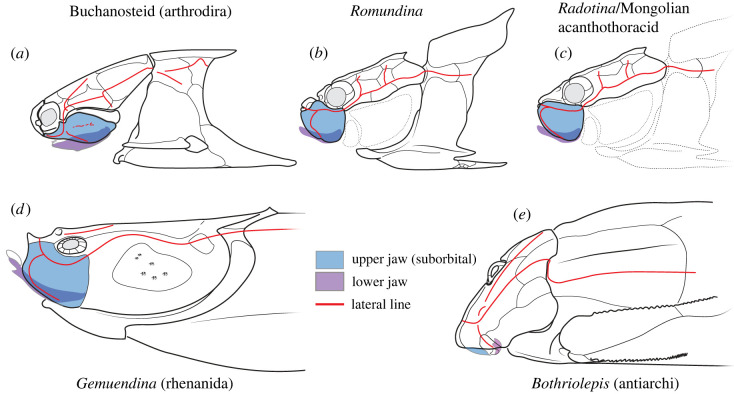
Cheek morphology and bite pattern in placoderm fishes with suborbital plates. (*a–d*) Placoderms displaying pronounced overbite with lower jaw contact on internal surface of upper jaw; (*e*) highly ventralized jaw mechanics of antiarchs for inferred ‘scraping’ feeding [42]. Illustration in (*a*) is original composited from [39,43,44]. Illustrations in (*b*) and (*c*) adapted from [45]; (*e*) adapted from [42,46]. Red lines indicate paths of the sensory line canals.

MPC-Fh200/10.4 allows us to accurately reconstruct the axis of jaw closing and the position of the bite in an ‘acanthothoracid’. To date, this has generally been inferred from fragmented and incompletely preserved fossils. Our material indicates that the lower jaw closed against the internal surface of the autopalatine, likely against a ridge that traverses this face (figure 3). This contrasts with the recent reconstruction of *Radotina,* which implied either that the bite was positioned on the labial margin of the suborbital plate or that an overbite of the submarginal was involved in prey capture or processing. Such a bite, where the suborbital carries the upper dentition, is anomalous compared with disparate other ‘placoderms’ such as rhenanids and arthrodires. In those taxa, the bite closes against the inner surface of the autopalatine and not the suborbital plate [34,39,41] (figure 3), regardless of their dramatically different craniofacial geometries (figure 4). These taxa are known from articulated specimens, or specimens in which the dental plates are intact. However, an alternative geometry is seen in *Bothriolepis,* where articulated specimens suggest the suborbital plate may have been involved in biting [40] superficially resembling the implied condition in *Radotina* [8]. Assessing the position of the bite in ‘acanthothoracids’ could help address which of these two ‘bite models’ is more appropriate.

The well-preserved quadrate of MPC-Fh200/10.4 allows us to reconstruct the orientation of the jaw hinge and mandible (electronic supplementary material, figure S2). As the quadrate is mediolaterally wide and nearly co-planar with the inner surface of the autopalatine, the lower jaw almost certainly closed against the latter surface. This is supported by taking a line perpendicular to the quadrate hinge axis, corresponding to a hypothetical lower jaw which intersects the centre of the adductor chamber and traverses the autopalatine surface. Thus, we consider the bite in ‘acanthothoracids’ inclusive of *Radotina* to have been located on the autopalatine, not the suborbital. This agrees with the general morphological similarities in overall structure between MPC-Fh200/10.4, *Radotina*, *Jagorina* and arthrodires (figures 3 and 4). The role of the submarginal plate in biting, ‘cleaver-shaped’ palatoquadrate, and small adductor fossa of *Bothriolepis* represent a clear anatomical outlier (figures 3 and 4) and has been attributed recently to ‘scraping’ feeding [42].

### 4.2. Implications for ‘placoderm’ dentitions

The new data from MPC-Fh200/10.4 show that the aligned tubercles interpreted as teeth in *Radotina* are not associated with the oral margin. The evidence for this is twofold: firstly, as noted above, these tubercles lie outside of a functional bite (figures 2–4). Second, we interpret the groove that these tubercles lie in as being for the supra-oral sensory line canal rather than the oral margin (figures 2 and 3). This feature was described by Vaškaninová *et al*. as a ‘trough with vascular foramina and grooves' and interpreted as the oral margin. MPC-Fh200/10.4 shows unambiguously that, instead, this is a sensory line canal connected to the infraorbital canal and facial innervation network. This is topologically identical to the same canal in *Romundina* (figure 2). The tubercles previously identified as teeth in *Radotina* lie dorsal to this supra-oral sensory line canal, and we propose that they are petal-shaped dermal tubercles. Petal-shaped tubercles with identical external morphology occur along the ventral margin of the infraorbital sensory canal in *Radotina* (see fig. S6 of the supplementary material of [8]), as well as elsewhere on the cheek. The comparative and functional anatomy above implies that the structures interpreted as teeth in *Radotina* could not have had a primary dental function and therefore have no direct bearing on the condition of teeth and jaw evolution in early gnathostomes.

### 4.3. Phylogenetic and evolutionary implications

A palatoquadrate fused to a dermal suborbital plate is a general condition of ‘placoderms’, as shown by numerous phylogenetically disparate examples (e.g. figures 3 and 4). Thus, this is very probably a primitive condition for jawed vertebrates. This is supported by our phylogenetic analysis, with this character (character 23) mapping to the ancestor of all jaw-bearing taxa. Although *Radotina* does not provide evidence of osteichthyan-like sutured dental plates in the mouths of ‘acanthothoracids’, Vaškaninová *et al*. convincingly demonstrate that tooth-bearing jaw bones were also more phylogenetically widespread in placoderms than previously understood. Indeed, our revised phylogenetic analysis supports an ancestral state of dermal bones borne on the jaws in jawed vertebrates (character 43). Coupled with the discoveries of Silurian ‘placoderms’ from China with osteichthyan-like jaw bones, there is an ever-growing case that heavily armoured jaws have an early phylogenetic origin. Although Vaškaninova *et al*. homologized the gnathal elements of placoderms with the dermal jaw bones of crown-group gnathostomes (e.g. premaxilla, maxilla and dentary), they did not re-code other placoderms for this new homology scheme. Nevertheless, this proposal was tested and further corroborated recently using a ‘dynamic homology’ approach [47]. If this is extrapolated to the origin of jaws themselves, it raises important evolutionary implications. Dermal-associated jaws provide a functionally informative model for the origins of jaws themselves. Up to now, debates on the origin of jaws have strongly emphasized the endoskeleton and questions of whether the palatoquadrate and hyoid arch represent derived gill arches of a branchiomeric ancestor. The absence of mineralized mandibular arches in fossil jawless fishes makes this scenario difficult to evaluate. This fact points to an overlooked problem: unmineralized endoskeletal cartilages are flexible and slightly elastic. Without support from the dermal skeleton, they would provide a structurally weak basis for the origin of jaws. We suggest that the phylogenetically deep association between exoskeletal and endoskeletal jaw presents a functionally plausible model.

## 5. Conclusion

The hypothesis that the ancestors of all jawed vertebrates possessed dermal jaw bones integrated with other facial jaw bones [9] is an important advance in our understanding of the origin of jaws. Dermal bone provides greater mechanical reinforcement than perichondrally ossified cartilage; it is therefore plausible that dermal bones provided a structural basis for jaws. As a purportedly primitive ‘placoderm’ assemblage, ‘acanthothoracids’ reveal jaw conditions with an important bearing on this hypothesis. Here we have shown that the morphology and function of ‘acanthoracid’ jaws resemble generalized ‘placoderm’ conditions seen also in arthrodires and rhenanids, consistent with past hypotheses [34]. Given the remote relationships between these assemblages, this indicates that jaw morphology was phylogenetically conserved across most ‘placoderms’. At present, there remains no evidence of an osteichthyan-like integration of tooth-bearing bones and dermal cheek plates deep in ‘placoderm’ evolution. A better understanding of jaw morphology in other ‘placoderms’, in conjunction with more stable phylogenetic hypotheses [5,6,19,38,48], will help to clarify these conditions further.

## Data Availability

The complete tomographic image data used in this analysis along with three-dimensional surface models can be accessed at: https://doi.org/10.6084/m9.figshare.20581665. The data are provided in the electronic supplementary material [49].
